# Time-Dependent Anchor Hole Expansion May Associate with Meniscal Extrusion After Open-Wedge High Tibial Osteotomy Combined with Medial Meniscus Posterior Root Tear Repair and Meniscal Centralization

**DOI:** 10.3390/bioengineering13020162

**Published:** 2026-01-29

**Authors:** Yohei Maeda, Ryuichi Nakamura, Kaori Matsumoto, Satomi Abe, Hiroshi Ito

**Affiliations:** 1 Department of Orthopedic Surgery, Joint Preservation and Sports Orthopedic Center, Harue Hospital, 65-7 Harue-cho, Haribara, Sakai 919-0476, Fukui, Japan; 2 Department of Orthopedic Surgery, Asahikawa Medical University, 2-1-1-1 Midorigaokahigashi, Asahikawa-shi 078-8510, Hokkaido, Japan; 3 Department of Radiological Technology, Harue Hospital, 65-7 Harue-cho, Haribara, Sakai 919-0476, Fukui, Japan

**Keywords:** medial meniscus posterior root tear, meniscus extrusion, high tibial osteotomy

## Abstract

**Background:** This study evaluated time-dependent changes in anchor hole width (AHW) and their association with postoperative medial meniscus extrusion (MME) in patients undergoing open-wedge high tibial osteotomy (OWHTO) with medial meniscus posterior root tear (MMPRT) repair and meniscal centralization. **Methods:** Thirty knees treated with combined OWHTO and MMPRT repair using the centralization technique were retrospectively reviewed. MRI, CT, and second-look arthroscopy were performed preoperatively and postoperatively. AHW of the MMPRT anchor and two centralization anchors (midbody and midbody–posterior, M-anchor and MP-anchor) were measured on multiplanar reconstruction CT images at 1, 3, and 6 months, and 1 year, and their correlations with postoperative MME were analyzed. **Results:** AHW increased up to 3 months and gradually decreased with surrounding sclerosis by 1 year. The M-anchor showed significantly greater mediolateral (ML) expansion than the MP-anchor and demonstrated a moderate positive correlation between 1-year AHW and MME (r ≈ 0.5, *p* < 0.01). Second-look arthroscopy confirmed a 90% healing rate of the repaired root. **Conclusions:** Although OWHTO combined with MMPRT repair and centralization achieved favorable root healing, postoperative MME progression was not fully prevented. Time-dependent ML anchor hole expansion around the M-anchor may indicate persistent micromotion, elongation of the meniscotibial ligament, and degenerative stretch of the repaired meniscus following healing, suggesting that even after successful root healing, ML motion remains difficult to control, highlighting the need for biomechanically optimized fixation.

## 1. Introduction

Disruption of the meniscal hoop function caused by a medial meniscus posterior root tear (MMPRT) leads to biomechanical changes comparable to those after total meniscectomy [1]. MMPRT has been recognized as a potential initiating factor for the progression of knee osteoarthritis (KOA) as well as for the development of subchondral insufficiency fracture of the knee (SIFK) and spontaneous osteonecrosis of the knee (SONK) [2].

MMPRT typically occurs in middle-aged to elderly patients, particularly women with early-stage osteoarthritis, and is often associated with degenerative changes in the medial compartment. When left untreated, MMPRT has a 5-year survival rate of 66% and a 10-year survival rate of 54%, with a high likelihood of requiring total knee arthroplasty (TKA) [3]. Furthermore, even after partial meniscectomy, approximately 90% of patients required TKA within 14 years [4].

Various repair techniques for MMPRT, including transtibial pullout repair and suture anchor repair, have been reported [5,6,7]. Transtibial pullout repair allows anatomic restoration of the meniscal root and has demonstrated favorable long-term outcomes; however, tunnel-related complications, such as tunnel widening, have been reported. In contrast, suture anchor repair eliminates the need for tibial tunnels and offers improved reproducibility, although it is associated with higher implant costs and technical challenges related to posterior portal placement [8].

Despite successful root repair, varus malalignment and obesity have been identified as poor prognostic factors for MMPRT repair [9,10]. Therefore, high tibial osteotomy (HTO) has been utilized to correct varus alignment and redistribute mechanical loading across the knee joint. By shifting the mechanical axis laterally, HTO reduces medial compartment stress and provides a favorable biomechanical environment for meniscal healing. Accordingly, combined treatment with HTO during MMPRT repair has been increasingly reported to improve clinical outcomes [11,12,13].

More recently, meniscal centralization has been introduced as an adjunctive procedure to MMPRT repair to address residual medial meniscus extrusion (MME). Several studies have demonstrated that adding centralization to MMPRT repair can reduce MME and improve clinical outcomes, such as Lysholm scores [14,15]. The centralization procedure repositions the extruded meniscus toward the tibial plateau to restore hoop tension and normalize load distribution, thereby enhancing the biological and mechanical stability of the repair construct. However, complete functional restoration of the meniscus remains challenging [16], and the effectiveness of the centralization technique has been reported to depend on factors such as anchor insertion site, insertion angle, and surrounding bone quality [17].

In addition, mechanical behavior at the bone–anchor interface may differ according to the deployment configuration of all-suture anchors, potentially influencing micromotion and subsequent bone responses. Although tunnel enlargement has been widely reported after anterior cruciate ligament (ACL) reconstruction [18,19], no studies have documented tunnel enlargement or anchor hole expansion in association with MMPRT repair. Furthermore, while anchor hole expansion has been reported in shoulder surgery, particularly after Bankart repair [20,21], to date, no studies have investigated anchor hole expansion in the knee following meniscal root repair.

We hypothesized that greater postoperative anchor hole expansion is significantly associated with increased MME in patients undergoing medial meniscus posterior root tear repair with meniscal centralization during open-wedge high tibial osteotomy (OWHTO).

## 2. Materials and Methods

### 2.1. Study Population

This retrospective cohort study was approved by the institutional review board of the authors’ institution (approval number: 2022-5; approval date: 20 September 2022). The study was conducted in accordance with the ethical standards of the Declaration of Helsinki. Because of the retrospective study design, the requirement for written informed consent was waived, and patient consent was obtained through an opt-out process.

This study included patients who underwent OWHTO between August 2020 and December 2022. The inclusion criteria were as follows: (1) OWHTO repair combined with the centralization technique performed concurrently with MMPRT repair [22,23,24], (2) minimum follow-up period of 2 years, (3) second-look arthroscopy performed at 1 year postoperatively, (4) MRI evaluation was performed preoperatively and at 1 year postoperatively following implant removal, and (5) CT evaluation performed at 1 month, 3 months, 6 months, and 1 year after surgery. Only patients who consented to implant removal, second-look arthroscopy, and repeated postoperative imaging were included in this study, resulting in a highly compliant and selected patient cohort.

Demographic data were obtained from electronic medical records, including patient age, sex, body mass index (BMI), Kellgren–Lawrence osteoarthritis grade (KL grade), correction angle, bone mineral density (BMD) of lumbar spine (L1–L4) T-score.

The inclusion criteria were intentionally restrictive to ensure methodological consistency and reliable longitudinal evaluation. Requirements for serial imaging, minimum follow-up duration, standardized surgical technique, and exclusion of advanced osteoarthritis were applied to minimize heterogeneity and to allow accurate assessment of time-dependent changes in anchor hole width and MME.

### 2.2. Surgical Indication

The indications for MMPRT repair combined with the centralization technique were as follows: (1) acute onset of MMPRT, (2) symptom duration within 6 months (preferably within 3 months), (3) MME less than 5 mm, including cases with mild or minimal extrusion, (4) absence of marked meniscal degeneration or thickening, and (5) no evidence of severe osteoporosis. In addition, the following criteria were applied for performing concurrent OWHTO: (1) weight-bearing line ratio (WBLR) < 50%, (2) mechanical medial proximal tibial angle (mMPTA) < 85°, and (3) history of surgical treatment for KOA, SIFK, or SONK secondary to MMPRT in the contralateral knee. Patients with mild or minimal MME were also included if the above criteria were satisfied. No upper age limit was predefined, and elderly patients were included provided that they had early to moderate knee osteoarthritis, defined as KL grade ≤ 3.

### 2.3. Surgical Technique

The surgical procedure was performed according to the methods described by Nakamura et al., with the techniques for OWHTO and MMPRT repair following their report [22], and the meniscal centralization procedure performed as described in their subsequent article [23].

Under general anesthesia, the patient was placed in the supine position, with the contralateral limb positioned lower than the operative limb to improve visualization of the medial compartment. An AssistArm positioner (CONMED, Largo, FL, USA) was attached to the operative side of the table to hold the leg during arthroscopy. After sterile preparation and draping, a reverse curved oblique skin incision was made to expose the site for OWHTO, as routinely performed in our institution. The upper border of the pes anserinus was incised while preserving its tibial attachment, and the superficial medial collateral ligament (sMCL) was completely released. These steps allowed maintenance of the hip abduction, knee flexion, and leg elevation positions without assistant traction, providing a wide posteromedial working space when using the posteromedial portal. The release of the sMCL also created sufficient medial joint space for root repair.

A standard diagnostic arthroscopy was performed through the anterolateral (ALP) and anteromedial portals (AMP) to evaluate the medial meniscus using a probe. A far anteromedial portal (FAMP) and the high mid-medial portal (HMMP) were then created just above the medial meniscus under arthroscopic guidance via the ALP. The posteromedial portal (PMP) was established under visualization using 30° and 70° arthroscopes (Synergy Vision System, Arthrex, Naples, FL, USA) inserted through the AMP.

For root repair, the native footprint of the posterior root was prepared using a radiofrequency ablation probe, curette, and motorized shaver inserted through the PMP. A 1.8 mm Q-Fix anchor (Smith & Nephew, Andover, MA, USA) was placed at the root attachment site through the PMP, and mattress sutures were created through the AMP. For the outer portion of the meniscus, a FIRSTPASS suture passer (Smith & Nephew) was used, whereas for the inner portion, a Scorpion suture passer (Arthrex) was utilized.

For the centralization procedure, the meniscocapsular attachment was adequately released from the tibial plateau using a rasp. Two 1.4 mm non-sliding JuggerKnot Soft Anchor Systems (Zimmer-Biomet, Warsaw, IN, USA) were inserted through the HMMP into the medial edges of the middle and posteromedial portions of the medial tibial plateau. A nylon loop mounted on an AcuPass suture passer (Smith & Nephew) was inserted through the AMP to penetrate the meniscus at the meniscocapsular junction. Mattress sutures were created using the suture relay technique, and the sutures were retrieved through the AMP.

**Figure 1:**
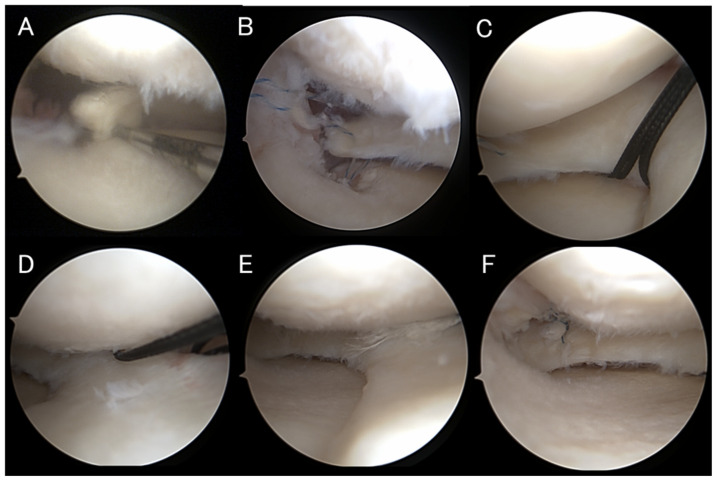
(**A**) Arthroscopic findings of a LaPrade type II A MMPRT [28]. (**B**) The posterior root was automatically reduced to its native attachment site, and a 1.8 mm Q-Fix anchor was placed at the root attachment site, and mattress sutures were created. (**C**) For centralization, two 1.4 mm non-sliding JuggerKnot Soft Anchor Systems were inserted at the medial edges of the mid- and posteromedial portions of the tibial plateau. (**D**) Mattress sutures were created using the suture relay technique, and the sutures were retrieved. (**E**,**F**) After releasing valgus stress to prevent meniscal elevation, the sutures were tied sequentially—first the centralization sutures, then the root repair sutures.

After releasing the valgus stress to prevent meniscal elevation from the tibial plateau, the centralization sutures were tied sequentially through the AMP without using a cannula. The sutures were passed in an anteroposterior direction with a non-sliding configuration to secure stable fixation. This initial tying automatically reduced the posterior root to its native attachment site. The root repair sutures were then tied through the AMP (Figure 1).

**Figure 2:**
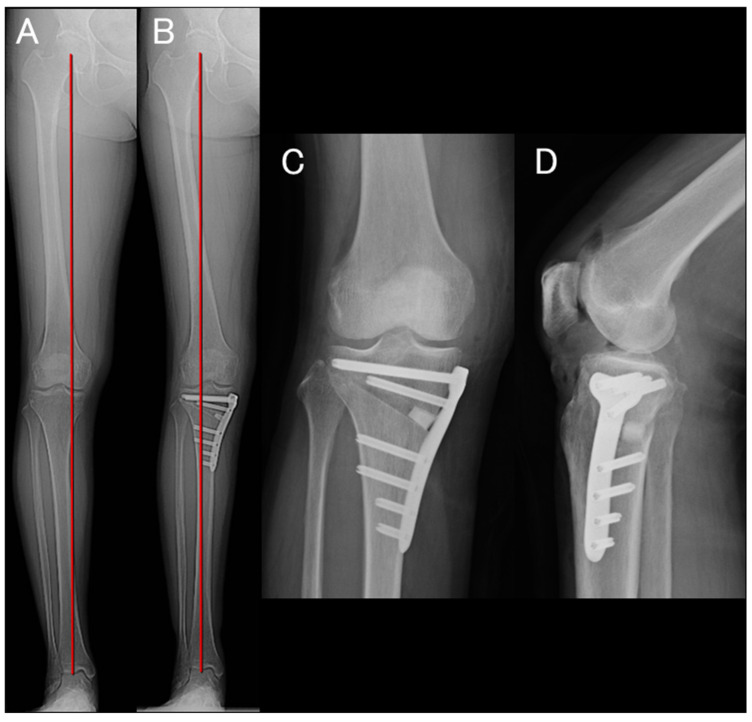
(**A**) Preoperative standing full-length anteroposterior radiograph of the lower extremity showing WBLR of 28.4%. (**B**) Postoperative standing full-length anteroposterior radiograph of the lower extremity showing WBLR of 63.4%. (**C**) Postoperative anteroposterior radiograph showing an 8 mm medial opening gap. (**D**) Postoperative lateral radiograph demonstrating that the bone substitute was inserted to provide firm posterior–medial support.

After completion of the arthroscopic procedure, OWHTO was performed in the same manner as in cases without meniscal repair [23,25]. Fixation was achieved using a TriS plate (OSferion Biomaterials, Tokyo, Japan), and the bone substitute was inserted into the osteotomy gap (OSferion 60 Marvelous, OSferion Biomaterials) (Figure 2). WBLR was adjusted to 58–72% of the tibial plateau width depending on the severity of osteoarthritis and cartilage condition [26,27].

### 2.4. Postoperative Rehabilitation

Range of motion (ROM) exercises were prohibited for the first 2 weeks after surgery. ROM exercises were initiated according to the postoperative protocol: knee flexion was permitted up to 90° at 2 weeks, up to 120° at 4 weeks, and without restriction after 6 weeks. Full weight-bearing was permitted immediately after surgery according to the postoperative protocol. The weight-bearing protocol was identical to that used for OWHTO without meniscal repair; however, the use of a knee brace was recommended at the initiation of weight-bearing to maintain knee extension.

### 2.5. Clinical and Radiographic Evaluation

(1) Radiographic assessments

Standing full-length anteroposterior radiographs of the lower extremity using slot-scan digital radiography and full-length anteroposterior radiographs of the tibia were obtained preoperatively and at 2 years postoperatively. The radiographic parameters measured included WBLR, mechanical lateral distal femoral angle (mLDFA), mMPTA, and joint line convergence angle (JLCA).

(2) MRI assessments

**Figure 3:**
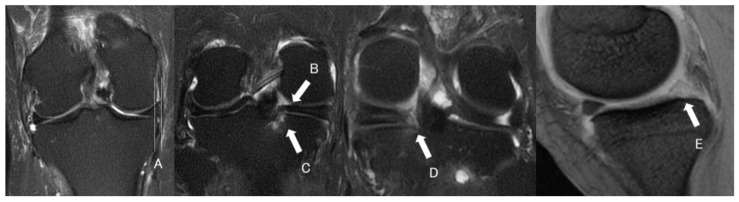
(A) Medial meniscus extrusion (MME) was measured at the center of the tibial plateau. Two vertical lines were drawn—one tangential to the medial edge of the tibial plateau and another parallel line along the outer edge of the medial meniscus—and the distance between the two lines was defined as MME. (B) A lateral view of a giraffe neck–like shape of the medial meniscus posterior segment on coronal MRI was defined as the giraffe neck sign. (C) Radial separation of the posterior root fibers resembling divergent roots was defined as the spreading roots sign. (D) An abrupt discontinuity of the posterior root contour was defined as the truncation sign. (E) Loss of the normal intrameniscal signal, appearing as a homogeneously white structure, was defined as the white meniscus sign.

MRI scans were performed preoperatively and at 1 year postoperatively using a 1.5-T scanner equipped with a dedicated knee coil (SIGNA Explorer, GE Healthcare, Hino, Japan). On coronal images, the MME distance was measured, and the presence of the giraffe neck sign [29], spreading roots sign [30], and truncation sign was evaluated. On sagittal images, the presence of the white meniscus sign was assessed (Figure 3). The prevalence of each finding was calculated. The standard imaging protocol included fat-suppressed T2-weighted sequences in the coronal plane and proton density fat-suppressed sequences in the sagittal plane, with a slice thickness of 3.0 mm. Intraobserver reliability of the MME measurements was assessed using the intraclass correlation coefficient (ICC). For all 30 knees, the same observer performed repeated measurements of MME after a 4-week interval. The intraobserver ICC for MME was 0.93, indicating excellent reliability. Interobserver reliability was also evaluated using the ICC between two independent observers. The interobserver ICC for MME was 0.91, likewise indicating excellent reliability.

(3) Clinical outcomes

Patient-reported outcomes were assessed using the Knee Injury and Osteoarthritis Outcome Score (KOOS). All subscales—pain, symptoms, activities of daily living (ADL), sport/recreation, and quality of life (QOL)—were evaluated preoperatively and at 2 years postoperatively. Additionally, knee extension and flexion angles were assessed preoperatively and at 2 years postoperatively.

(4) Second-look arthroscopy

**Figure 4:**
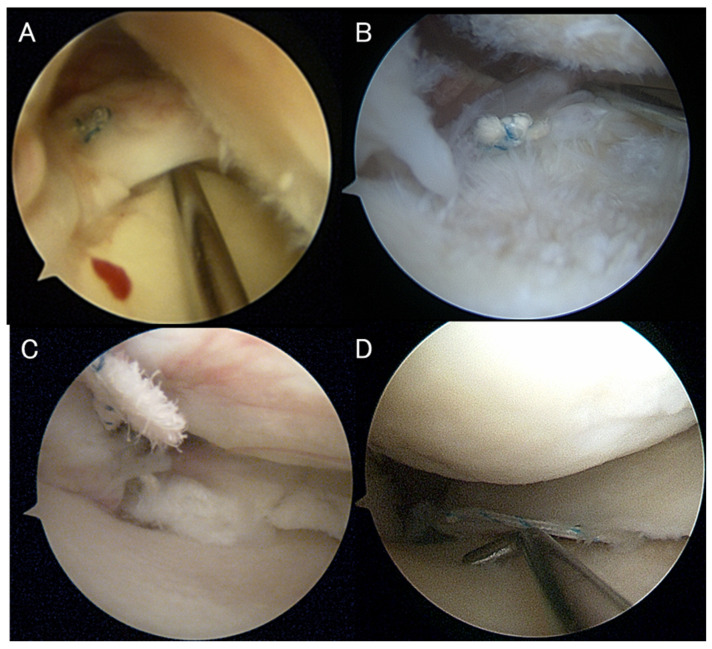
Second-look arthroscopic classifications of MMPRT healing, defined according to the criteria of Seo et al. [31]. Four representative arthroscopic images illustrate the various types of healing. (**A**) Complete healing was defined as a condition in which the repaired meniscus was anatomically reduced and firmly reattached to its native footprint. (**B**) Lax healing was characterized by an almost normal meniscal appearance that was easily lifted by probing, although continuity was preserved. (**C**) Scar healing was defined as a superficially healed appearance in which only fibrous scar tissue connected the tibial attachment to the posterior horn, lacking true meniscal continuity. (**D**) Failed healing was defined as the absence of continuity at the repaired site, with no evidence of healing observed.

Second-look arthroscopy was performed at the time of OWHTO plate removal, approximately 1 year after surgery, to evaluate healing of the MMPRT. The healing status of the medial meniscus posterior root was classified according to the criteria described by Seo et al. into complete healing, lax healing, scar healing, and failed (Figure 4) [31]. The proportion of each healing type was also calculated.

(5) Multiplanar reconstruction (MPR)-CT imaging assessments

**Figure 5:**
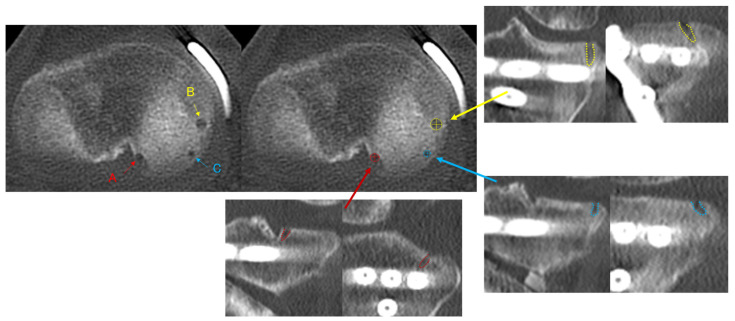
CT images showing anchor holes and anchor hole width (AHW) for each anchor: (A) the anchor hole for the anchor used in MMPRT repair (R-anchor), (B) the anchor hole for the midbody anchor (M-anchor) used in the centralization technique, and (C) the anchor hole for the midbody–posterior anchor (MP-anchor). The maximum anteroposterior and mediolateral AHW (AP-AHW and ML-AHW) were measured.

Multiplanar reconstructed computed tomography (MPR-CT) imaging (SCE-NARIA View; FUJIFILM, Tokyo, Japan) was performed at 1, 3, and 6 months, and at 1 year postoperatively. CT imaging was conducted not only to quantitatively evaluate time-dependent changes in anchor hole width (AHW) but also for the clinical assessment of bone healing after OWHTO. CT was considered essential because subtle morphological changes in AHW, as well as bone healing at the osteotomy site, cannot be reliably assessed using plain radiographs or magnetic resonance imaging due to insufficient spatial resolution. All CT examinations were performed using a predefined low-dose imaging protocol to minimize radiation exposure; the volume CT dose index (CTDIvol) for each scan was <5 mGy, which is substantially lower than that of standard diagnostic knee CT protocols. The repeated CT imaging protocol was justified by both research and clinical purposes and was approved by the institutional review board. The AHW was evaluated for the anchor used for MMPRT repair (root anchor; R-anchor), the centralization anchor placed from the midbody to the slightly posterior region of the meniscus (midbody anchor; M-anchor), and the anchor placed at the midbody–posterior transition zone (midbody–posterior anchor; MP-anchor) (Figure 5). The maximum anteroposterior and mediolateral AHW (AP-AHW and ML-AHW) were measured on MPR-CT images. The postoperative 1-month AHW was defined as the initial reference value (baseline), and subsequent changes in AHW at 3 months, 6 months, and 1 year postoperatively were evaluated relative to this baseline. The slice thickness of the MPR-CT images was 0.625 mm. Intraobserver reliability of the measurements was assessed using the ICC. For all 30 knees, the same observer performed repeated measurements of the AP-AHW and ML-AHW for each anchor after a 4-week interval. The intraobserver ICCs ranged from 0.91 to 0.92 for the R-anchor, 0.92 to 0.93 for the M-anchor, and 0.90 to 0.92 for the MP-anchor, indicating excellent reliability. Interobserver reliability was also evaluated using the ICC between two independent observers. The interobserver ICCs ranged from 0.88 to 0.90 for the R-anchor, 0.87 to 0.89 for the M-anchor, and 0.88 to 0.89 for the MP-anchor, likewise indicating excellent reliability.

### 2.6. Statistical Analysis

All statistical analyses were conducted including all 30 knees without exclusion based on postoperative healing status. Knees with incomplete or failed MMPRT healing were retained in the analysis in accordance with an intention-to-observe approach. Radiographic assessments, MRI data, clinical outcomes, and MPR-CT imaging data were compared between preoperative and postoperative values, as well as among postoperative time points. Paired t-tests were used to compare continuous variables between preoperative and postoperative measurements, including WBLR, mLDFA, mechanical mMPTA, JLCA, MME, KOOS subscales (Pain, Symptoms, Activities of Daily Living, Sport/Rec, and Quality of Life), and knee extension and flexion angles. Longitudinal changes in AHW were analyzed using linear mixed-effects models to account for within-subject correlations arising from repeated measurements appropriately. Separate models were constructed for each anchor location (M-anchor, MP-anchor, and R-anchor) and for each measurement direction (ML and AP). Time after surgery (1, 3, 6, and 12 months) was included as a fixed effect, and patient was included as a random effect with a random intercept. Time was treated as a categorical variable, and post hoc pairwise comparisons between time points were performed using estimated marginal means with Bonferroni adjustment. For comparisons between anchor locations, AHW at the M-anchor was additionally compared with that at the MP-anchor. Changes in the prevalence of MRI findings were analyzed using the McNemar test for paired binary data. Pearson’s correlation coefficients were calculated to evaluate the relationships between postoperative MME and AHW, as well as between AHW and demographic variables. Univariate analyses were first performed to assess the association between postoperative MME and each AHW parameter individually. Subsequently, multivariable linear regression analyses were conducted to determine whether AHW parameters were independently associated with postoperative MME after adjustment for potential confounders. To avoid multicollinearity among anchor-related variables, separate multivariable models were constructed for each anchor (M-, MP-, and R-anchor) and for each direction (ML and AP). All multivariable models were adjusted for age, body mass index, correction angle, bone mineral density, postoperative WBLR, postoperative mMPTA, postoperative mLDFA, and postoperative JLCA. Given the relatively small sample size, these multivariable analyses were regarded as exploratory and hypothesis-generating rather than confirmatory. Results are presented as estimated marginal means with standard deviations unless otherwise stated. All statistical analyses were performed using Python (version 3.10; Python Software Foundation, Wilmington, DE, USA), and statistical significance was defined as a two-tailed *p* value < 0.05.

## 3. Results

Among the 228 knees that underwent OWHTO during the study period, 30 knees met the eligibility criteria. The demographic data of the included patients are shown in Table 1. Although several patients were classified as having osteoporosis based on T-scores, none had severe osteoporosis associated with fragility fractures.

Preoperative and 2-year postoperative radiographic and MRI assessments demonstrated significant improvements in WBLR and mMPTA (both *p* < 0.001), whereas JLCA showed no significant change (*p* = 0.34). MME, however, significantly increased postoperatively (*p* < 0.001). All KOOS subscales—pain, symptoms, ADL, sport/recreation, and QOL—showed significant improvements at 2 years postoperatively (all *p* < 0.001). The calculated minimal clinically important difference (MCID) values based on the distribution method (0.5 × SD of score change) were 10.4 for Pain, 11.3 for Symptoms, 8.7 for ADL, 14.1 for Sport/Rec, and 12.1 for QOL. The achievement rates for MCID were 93.3%, 56.7%, 73.3%, 73.3%, and 86.7% for Pain, Symptoms, ADL, Sport/Rec, and QOL, respectively. Most patients achieved clinically meaningful improvement, particularly in the Pain and QOL domains. Knee extension angle improved significantly (*p* < 0.001), while flexion angle showed no significant change (*p* = 0.12) (Table 2).

Regarding MRI findings, the prevalence of the giraffe neck sign, spreading roots sign, truncation sign, and white meniscus sign all significantly decreased at 1 year postoperatively (*p* < 0.001) (Table 3). At the time of plate removal, approximately 1 year after surgery, second-look arthroscopy revealed that the overall healing rate of MMPRT, including complete, lax, and scar healing, was 90.0% (Table 4).

**Figure 6:**
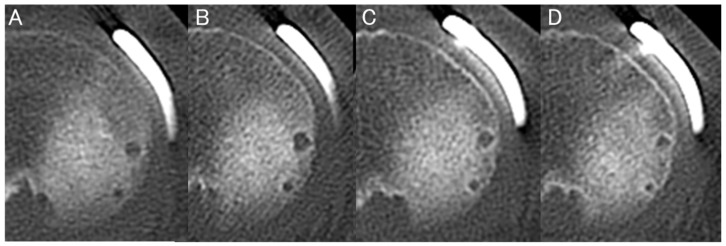
Representative serial changes in AHW observed on MPR CT images at 1 month (**A**), 3 months (**B**), 6 months (**C**), and 1 year (**D**) postoperatively. AHW increased and peaked at approximately 3 months, then gradually decreased with surrounding bone sclerosis by 6 months to 1 year postoperatively.

Representative serial MPR CT images obtained at 1, 3, 6, and 1 year postoperatively are shown in Figure 6. AHW increased at 1 month, reached its maximum at approximately 3 months, and subsequently decreased with surrounding sclerosis by 6 months to 1 year. At the R-anchor, both ML-AHW and AP-AHW showed a decreasing trend over time. AP-AHW decreased significantly from 2.4 ± 0.8 mm at 1 month to 1.9 ± 1.1 mm at 1 year (*p* = 0.03), whereas changes in ML-AHW did not reach statistical significance (*p* = 0.09). At the M-anchor, ML-AHW demonstrated a significant time-dependent increase according to the linear mixed-effects model (*p* < 0.001), increasing from 2.7 ± 0.7 mm at 1 month to 3.5 ± 1.0 mm at 3 months, 3.3 ± 1.1 mm at 6 months, and 3.1 ± 1.1 mm at 1 year. Post hoc analyses revealed that ML-AHW at 3, 6 months, and 1 year was significantly larger than that at 1 month, whereas no significant difference was observed between 6 months and 1 year. In contrast, AP-AHW at the M-anchor did not show a significant time effect (*p* = 0.18), remaining relatively stable over time (2.8 ± 0.8 mm at 1 month and 3.4 ± 1.3 mm at 1 year). At the MP-anchor, ML-AHW also increased significantly over time (*p* = 0.004), from 2.3 ± 0.6 mm at 1 month to 3.0 ± 0.8 mm at 3 months, 2.9 ± 0.9 mm at 6 months, and 2.7 ± 1.0 mm at 1 year, although the magnitude of change was smaller than that observed at the M-anchor. No significant time-dependent change was observed in AP-AHW at the MP-anchor (*p* = 0.27) (Table 5). Additionally, no significant differences in AP-AHW were found between the M-anchor and MP-anchor throughout the postoperative period. Conversely, ML-AHW was significantly larger for the M-anchor than for the MP-anchor at all postoperative time points (*p* < 0.05) (Table 6).

**Figure 7:**
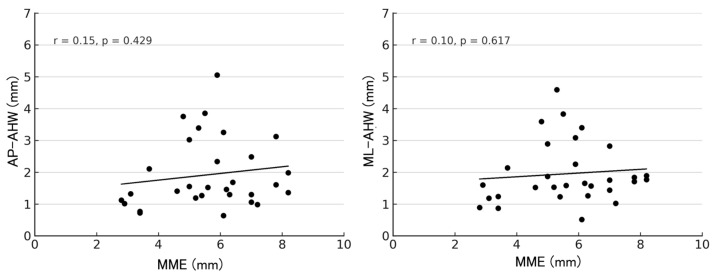
Correlation between MME and AHW of the R- anchor at 1 year postoperatively. AP-AHW: r = 0.15, *p* = 0.429; ML-AHW: r = 0.10, *p* = 0.617.

**Figure 8:**
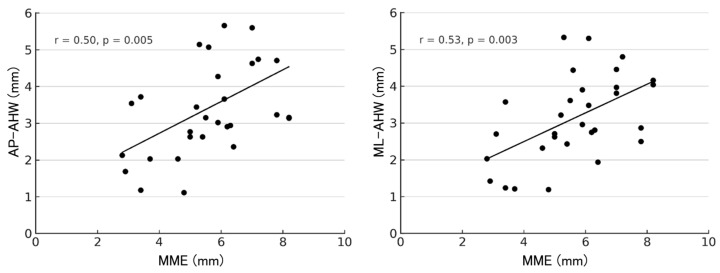
Correlation between MME and AHW of the M-anchor at 1 year postoperatively. AP-AHW: r = 0.50, *p* = 0.005; ML-AHW: r = 0.53, *p* = 0.003.

**Figure 9:**
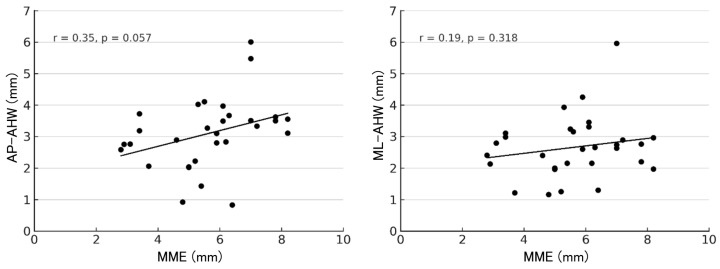
Correlation between MME and AHW of the MP-anchor at 1 year postoperatively. AP-AHW: r = 0.35, *p* = 0.057; ML-AHW: r = 0.19, *p* = 0.318.

At 1 year postoperatively, correlations between MME and the AHW of each anchor are shown in Figure 7, Figure 8 and Figure 9. For the R-anchor, AP-AHW (r = 0.15, *p* = 0.429) and ML-AHW (r = 0.10, *p* = 0.617) showed no significant correlations. In contrast, for the M-anchor, AP-AHW (r = 0.50, *p* = 0.005) and ML-AHW (r = 0.53, *p* = 0.003) both showed significant moderate positive correlations with MME. For the MP-anchor, AP-AHW (r = 0.35, *p* = 0.057) demonstrated a weak, non-significant trend, and ML-AHW (r = 0.19, *p* = 0.318) showed no significant correlation.

Finally, univariate analyses demonstrated significant associations between postoperative medial meniscus extrusion and both mediolateral and anteroposterior anchor hole expansion at the M-anchor. In contrast, no significant associations were observed for the MP- or R-anchor. In multivariable linear regression analyses adjusting for patient-related, bone-related, and postoperative alignment parameters, both mediolateral and anteroposterior anchor hole expansion at the M-anchor remained independently associated with postoperative medial meniscus extrusion (Table 7). These findings indicate that the observed associations at the M-anchor were not explained by patient characteristics or postoperative alignment factors.

## 4. Discussion

The most important finding of this study was that, despite achieving a high healing rate of the repaired root after combined OWHTO, MMPRT repair, and meniscal centralization, postoperative progression of MME was still observed. Moreover, a moderate correlation was found between M-anchor hole expansion and the degree of MME. These results suggest that even after successful root healing and centralization, the repaired meniscus may gradually extrude due to micromotion around the anchor under load-bearing conditions. This may be attributed to elongation of the meniscotibial ligament or intrinsic meniscal generative stretch. The time-dependent increase in AHW observed around the M-anchor likely reflects these micromotions. Although significant associations were observed, these findings do not imply causality and should be interpreted as hypothesis-generating, given the relatively small sample size and the exploratory nature of the multivariable analyses.

In addition, both the M-anchor and MP-anchor showed significant expansion of AHW in the ML direction, whereas no significant expansion was observed in the AP direction. This finding suggests that subtle ML micromotion of the repaired meniscus persisted even after AP stabilization had been achieved through root healing. This interpretation is consistent with the findings of Shimozaki et al. [32], who reported that in early KOA, the medial meniscus exhibited significantly greater ML displacement than in healthy knees, regardless of loading condition. Furthermore, they found that although ML displacement was not significantly affected by the presence of MMPRT, posterior displacement was significantly greater in knees with MMPRT, indicating that MMPRT mainly influences posterior extrusion. These findings suggest that the meniscus undergoes dynamic elongation and shortening during loading, contributing to its shock-absorbing function. Therefore, because MMPRT was successfully repaired and AP stability was restored, no significant difference in AP-AHW expansion was observed between the centralization anchors; however, persistent ML micromotion associated with the centralization anchors likely remained and contributed to anchor hole expansion at the M-anchor, as well as to the progression of MME. If the MMPRT repair had failed, additional posterior extrusion would likely have occurred due to loss of root integrity. However, the observed association between medial anchor hole expansion and postoperative medial meniscus extrusion should be regarded as a finding derived from this selected elderly cohort. While this association is intriguing and clinically relevant, it does not imply causality. Given the relatively small sample size of 30 knees, the observed association should be interpreted with caution and regarded not as conclusive evidence of causality, but rather as hypothesis-generating observations that highlight a potential structural mechanism contributing to residual or progressive extrusion despite apparently successful root healing.

Previous studies have employed relatively aggressive postoperative rehabilitation protocols following MMPRT repair combined with OWHTO. Koga et al. [33] allowed unrestricted range of motion immediately after surgery, with partial weight-bearing initiated early and full weight-bearing permitted at 6 weeks, whereas Mochizuki et al. [34] initiated range of motion at 1 week postoperatively with non–weight-bearing for the first 2 weeks and full weight-bearing at 4 weeks. Katagiri et al. [35] adopted an even more accelerated protocol, permitting range of motion from the day after surgery, partial weight-bearing from postoperative day 3, and full weight-bearing as early as 2 weeks postoperatively. In the present study, an early postoperative weight-bearing protocol was employed, and mechanical stress may have been transmitted to the anchors and the surrounding bone–meniscal or meniscocapsular interface before sufficient biological healing had occurred. Although OWHTO shifts the weight-bearing axis laterally and reduces loading of the medial compartment [36], these early loading conditions may nevertheless have induced subtle micromotion at the anchor–bone interface. Such micromotion may have contributed to anchor hole expansion and progressive medial meniscus extrusion, even in cases in which root healing appeared to be successful. Therefore, while early postoperative weight-bearing is beneficial for functional recovery, it may represent a potential mechanical risk factor for residual or progressive meniscal extrusion through anchor-related micromotion, particularly in elderly patients with compromised bone quality.

**Figure 10:**
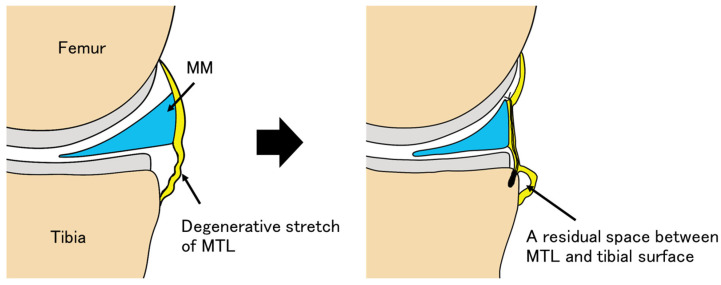
Meniscal centralization is a procedure that does not directly restore or heal degenerative stretching of the meniscus itself, but instead involves plication of the elongated meniscotibial ligament (MTL). Although the bone–capsular junction is debrided with a rasp and fixed to the tibial rim using an anchor, a residual interface between the meniscotibial ligament and the tibial surface may remain. Under this hypothetical model, early postoperative loading may induce micromotion at the anchor–bone interface, and this anchor micromotion may secondarily result in elongation of the meniscotibial ligament and degenerative stretch of the meniscocapsular complex. Over time, these processes may contribute to incomplete biological integration and progressive meniscal extrusion. MM, medial meniscus.

Regarding the underlying mechanism, Yamakado et al. [37] reported in anterior cruciate ligament reconstruction that the entrance of the bone tunnel, which is subjected to tensile stress, showed lamellar bone formation at 12 weeks and tendon–bone junction formation at 6 months, whereas healing within the tunnel itself remained incomplete. Although this healing pattern was observed in a different anatomical and biomechanical context, a similar biological concept may be relevant to MMPRT repair in the present study. From a mechanistic perspective, we hypothesize that anatomical anchor placement during MMPRT repair directly restores the bone–meniscal junction, which may facilitate earlier biological integration and limit micromotion at the repair site, thereby potentially reducing anchor hole expansion. In contrast, meniscal centralization primarily involves plication of the elongated meniscotibial ligament rather than direct restoration of the meniscal root attachment. Even though the bone–capsular junction was debrided and fixed to the tibial rim using an anchor, a residual interface between the meniscotibial ligament and the tibial surface may persist. Under this hypothetical model, such incomplete attachment may be less resistant to early postoperative loading, leading to micromotion at the anchor–bone interface and gradual degenerative stretch of the meniscocapsular complex. Over time, these processes could contribute to incomplete biological integration and progressive medial meniscus extrusion (Figure 10). Importantly, these mechanistic interpretations are speculative and hypothesis-generating, as the present study did not directly assess micromotion, tissue integration, or histological healing at the anchor–bone interface.

To overcome this limitation, the arthroscopic belt capsulodesis technique described by Nakayama et al. [38], which is designed to promote healing of the elongated meniscotibial ligament, may be beneficial. In this minimally invasive procedure, two knotless soft anchors are inserted along the medial tibial rim from the posteromedial corner toward the midbody, and sutures are passed in a belt-like configuration to pull and secure the meniscocapsular complex toward the tibial edge. This centralization-type technique allows re-centralization of the extruded meniscus and may effectively prevent further extrusion.

In addition, the possibility of intrinsic meniscal degenerative stretch should also be considered as a contributing factor to MME. Kita et al. [39] introduced an arthroscopic meniscal repair technique incorporating circumferential fiber augmentation to reinforce the circumferential fibers of the meniscus. This technique, which is biomechanically rational as a method to strengthen the hoop structure and prevent degenerative stretching of the meniscus, involves passing additional sutures along the circumferential fibers to compress the meniscus against the capsule, thereby reconstructing hoop tension and enhancing fixation strength. Biomechanical testing demonstrated significantly higher ultimate failure load and stiffness compared with the conventional method, suggesting that augmentation of circumferential fibers improves mechanical stability and may reduce postoperative retear and extrusion. Similarly, Morito et al. [40] described an arthroscopic “meniscal hoop-plasty” technique using a 1 mm polyethylene tape to reconstruct the circumferential hoop tension of the lateral meniscus. Their method enables anatomical restoration of hoop stress by circumferentially passing the tape through the red-red zone from the posterior to the anterior root and anchoring it via tibial bone tunnels. The technique provides full-circumference mechanical support while maintaining physiological meniscal mobility, potentially preventing or correcting meniscal extrusion. Early clinical and MRI observations showed reduction in lateral meniscus extrusion, indicating that this approach may offer a biomechanically robust and minimally invasive option for restoring hoop function. Biomechanical testing demonstrated significantly higher ultimate failure load and stiffness compared with the conventional method, suggesting that augmentation of circumferential fibers improves mechanical stability and may reduce postoperative retear and extrusion.

Although the pathology differs, a comparable concept can be found in arthroscopic Bankart repair for shoulder instability, which also employs all-suture anchors. Anchor hole expansion has been reported even in this non-weight-bearing joint [20,21]. Ruiz et al. [20] reported that among 72 Iconix 1.4 mm (Stryker, Kalamazoo, MI, USA) and 71 SutureFix 1.7 mm (Smith & Nephew, Watford, UK) anchors, 30.1% demonstrated expansion exceeding the drill diameter. While Bankart repair involves reattachment of the labrum–capsule complex rather than meniscotibial structures, the occurrence of anchor hole expansion in the shoulder underscores that similar expansion in centralization is unsurprising, particularly under load-bearing conditions, and may plausibly contribute to MME progression.

Furthermore, Lee et al. [21] investigated the relationship between anchor design and tunnel expansion after arthroscopic Bankart repair using four different anchor types (1.3-mm spherical, 1.4-mm cloverleaf, 1.7-mm omega-shaped, and 1.4-mm cylindrical). They reported mean tunnel diameters of 3.9, 3.3, 3.7, and 2.0 mm, respectively, with spherical anchors demonstrating the greatest degree of tunnel expansion. Despite these morphological differences, no significant differences were observed in postoperative clinical outcomes or healing rates. This finding indicates that anchor design can influence tunnel expansion even in non-weight-bearing joints such as the shoulder. Based on these observations, the deployment configuration of all-suture anchors may be an important factor determining both the direction and magnitude of micromotion at the bone–anchor interface. Spherical anchors expand radially and are therefore prone to multidirectional micromotion, which may result in generalized anchor hole expansion. In contrast, omega-shaped anchors preferentially expand in the mediolateral direction, which may explain selective ML-AHW even when anteroposterior stability is preserved. Cylindrical anchors have the smallest expansion diameter and may limit mediolateral and anteroposterior micromotion; however, they may be more susceptible to longitudinal micromotion along the anchor axis. While this characteristic may be advantageous in limiting ML or AP anchor hole expansion, repeated axial micromotion could manifest radiographically as deeper or elongated tunnel formation. Importantly, the meniscus is subjected to forces not only in the mediolateral and anteroposterior directions but also along the loading axis. Therefore, selecting a single anchor design that optimally accommodates all directions of mechanical loading remains challenging. In the present study, an omega-shaped 1.8-mm Q-FIX anchor was used for MMPRT repair, whereas two spherical 1.4-mm JuggerKnot Soft Anchor System anchors were used for meniscal centralization. Accordingly, differences in anchor configuration and diameter should be considered when interpreting anchor hole expansion. Because this was a retrospective analysis of a selected cohort, selection bias cannot be excluded, and further studies are warranted to determine the optimal anchor configuration for load-bearing joints such as the knee.

From a clinical perspective, however, structural changes in MME do not necessarily translate into short-term symptom deterioration. Previous studies have reported that changes in MME after OWHTO do not necessarily correlate with short-term clinical outcomes. Bae et al. [41] demonstrated that although MME changed serially after surgery, its progression or residual extrusion did not significantly affect patient-reported outcomes or arthroscopic cartilage improvement during short-term follow-up. These findings suggest that, while MME reflects structural disruption of the knee joint, its temporal behavior does not necessarily coincide with improvements in clinical symptoms such as pain relief and functional recovery observed in the early postoperative period after OWHTO. In other words, in the short term, clinical improvement is largely driven by unloading of the medial compartment through alignment correction and lateralization of the weight-bearing line, and residual or progressive structural meniscal extrusion may not immediately manifest as clinical deterioration.

Regarding MMPRT healing and MME following combined MMPRT repair and OWHTO, Kim et al. [12] reported that after pull-out repair using the modified Mason–Allen technique combined with OWHTO, MRI evaluation showed complete healing in 64.7% and incomplete healing in 29.4% of cases, whereas MME significantly increased from 3.0 mm preoperatively to 3.1 mm postoperatively. Similarly, Okamura et al. [13] demonstrated that pull-out MMPRT repair combined with OWHTO resulted in complete healing in 30.8% and partial healing in 57.1% of knees, with MME significantly increasing from 4.3 mm to 5.5 mm. In contrast, Tokumoto et al. [42] reported that combined centralization and OWHTO achieved complete healing in 80.6% and incomplete healing in 11.1% of cases, with postoperative improvement in MME observed in 83.3% of knees. In the present study, although combined MMPRT repair and centralization with OWHTO resulted in postoperative progression of MME, second-look arthroscopy demonstrated a high MMPRT healing rate, comparable to previous reports. These findings suggest that postoperative progression of MME was not attributable to poor MMPRT healing but may instead be associated with persistent micromotion and degenerative stretch of the repaired meniscus within the load-bearing joint, including mediolateral movement. Nevertheless, in the absence of centralization, even greater extrusion might have occurred. Centralization at the time of surgery likely facilitated anatomical reduction in the meniscal root, and, assuming that it provided temporary mechanical stability until anchor hole expansion occurred, it may have contributed to an improved healing rate of the repaired root. These findings suggest that centralization plays a biomechanically supportive role; therefore, further investigations are warranted to clarify the optimal fixation strategy for maintaining meniscal position under mechanical load.

Several limitations should be acknowledged. First, this was a single-center retrospective study with a small sample size and a relatively short follow-up period (1–2 years), which may not have been sufficient to fully evaluate long-term bone remodeling or the progression of meniscal extrusion. In particular, the limited sample size may have reduced the statistical power to detect moderate associations, especially for anchors other than the medial anchor; therefore, some findings that did not reach statistical significance cannot exclude the possibility of type II error. In addition, the study cohort comprised a highly selected group of elderly patients with early to moderate osteoarthritis (KL grade ≤ 3) who met strict inclusion criteria, including repeated CT imaging and second-look arthroscopy. Because only patients who consented to implant removal, second-look arthroscopy, repeated postoperative imaging, and long-term follow-up were included, selection bias cannot be excluded, and the cohort likely represents a highly compliant subgroup. This selection process further limits the generalizability of the findings, which should be interpreted within this specific clinical context and regarded as exploratory rather than definitive. Moreover, the combined treatment of OWHTO with MMPRT repair and meniscal centralization evaluated in this study should not be considered a widely applicable alternative to unicompartmental knee arthroplasty (UKA) or TKA, but rather a joint-preserving procedure for carefully selected patients. Furthermore, although AHW was evaluated serially using CT at 1, 3, and 6 months and at 1 year postoperatively, magnetic resonance imaging for assessment of MME was performed only at the 1-year time point. Therefore, the temporal relationship between changes in AHW and progression of MME could not be determined, and it remains unclear whether anchor hole expansion preceded meniscal extrusion or vice versa. In addition, the requirement for multiple CT examinations to assess AHW may limit the broad applicability of this evaluation strategy in routine clinical practice due to concerns regarding radiation exposure. Second, only one type of all-suture anchor was used for each fixation site, and the study did not include a control group without meniscal centralization or comparisons across different anchor configurations, insertion angles, or anchor types. In addition, the number of anchors used for MMPRT repair and meniscal centralization was fixed in all cases, and the potential influence of anchor number on fixation stability, micromotion, anchor hole expansion, or postoperative meniscal extrusion could not be evaluated. Therefore, the independent effects of anchor-related variables on anchor hole expansion and medial meniscus extrusion could not be determined. Third, comparisons with modified centralization techniques, such as circumferential fiber augmentation or capsulodesis, were not performed, and thus the influence of alternative surgical strategies on postoperative outcomes remains unclear. In addition, the early postoperative weight-bearing protocol used in this study may have influenced anchor behavior and anchor hole expansion, and this potential effect cannot be completely excluded. Finally, direct biomechanical evaluation of anchor fixation strength and histological confirmation of bone ingrowth were not conducted.

## 5. Conclusions

OWHTO combined with MMPRT repair and centralization achieved favorable root healing; however, postoperative progression of MME could not be completely prevented. Time-dependent ML AHW expansion around the M-anchor may reflect persistent micromotion, elongation of the meniscotibial ligament, and degenerative stretch of the repaired meniscus despite successful root healing. These findings suggest that, while root repair and centralization effectively restore posterior stability, complete control of ML motion and prevention of MME progression remain clinical challenges, highlighting the need for biomechanically optimized fixation.

## Figures and Tables

**Table 1:** Demographic data of the 30 knees included in this study.

Characteristic	
Age (year)	68.9 ± 7.6 (42 to 78)
Male/Female	2/28
Body mass index (kg/m^2^)	24.5 ± 3.7 (17.3 to 33.7)
KL grade 1/2/3/4	13/16/1/0
Correction angle (°)	8.2 ± 1.4 (5.0 to 10.0)
Bone mineral density of L1-4 T-score	−0.8 ± 1.2 (−2.7 to 3.0)

All data are presented as mean ± standard deviation (range), unless otherwise indicated.

**Table 2:** Comparison of preoperative and 2-year postoperative radiographic, MRI, and clinical parameters.

	Preoperative	Postoperative	*p*-Value
WBLR (%)	34.4 ± 6.9 (20.9 to 50.0)	61.1± 7.6 (39.5 to 79.3)	<0.001 *
mLDFA (°)	87.7 ± 1.6 (84.7 to 91.6)	88.0 ± 1.4 (86.2 to 92.1)	0.44
mMPTA (°)	85.0 ± 1.6 (82.1 to 88.4)	91.7 ± 2.3 (87.6 to 97.4)	<0.001 *
JLCA (°)	0.4 ± 1.5 (−1.4 to 4.6)	0.8 ± 1.7 (−2.7 to 4.0)	0.34
MME (mm)	3.7 ± 0.8 (1.6 to 4.8)	5.6 ± 1.5 (2.8 to 8.2)	<0.001 *
KOOS pain	55.8 ± 17.4 (21.4 to 91.7)	88.6 ± 10.0 (64.3 to 100)	<0.001 *
KOOS symptoms	65.0 ± 15.6 (32.1 to 100)	85.2 ± 9.2 (64.3 to 100)	<0.001 *
KOOS ADL	65.6 ± 15.2 (36.8 to 95.6)	88.2 ± 8.7 (69.1 to 100)	<0.001 *
KOOS sport/rec	30.5 ± 24.4 (0 to 75)	62.8 ± 18.6 (15 to90)	<0.001 *
KOOS QOL	30.0 ± 18.7 (0 to 81.3)	71.1 ± 14.6 (43.8 to 100)	<0.001 *
Knee extension angle (°)	−3.0 ± 3.6 (−10 to 0)	−0.6 ± 1.6 (−5 to 0)	<0.001 *
Knee flexion angle (°)	137.8 ± 8.8 (120 to 150)	141.0 ± 6.5 (130 to 150)	0.12

All data are presented as mean ± standard deviation (range), unless otherwise indicated. WBLR, weight-bearing line ratio; mLDFA, mechanical lateral distal femoral angle; mMPTA, mechanical medial proximal tibial angle; JLCA, joint line convergence angle; MME, medial meniscus extrusion; KOOS, Knee Injury and Osteoarthritis Outcome Score. * Significant improvement compared with preoperative value (*p* < 0.05).

**Table 3:** Comparison of the prevalence of positive MRI findings (giraffe neck sign, truncation sign, spreading roots sign, white meniscus sign) between preoperative and 1-year postoperative evaluations.

	Preoperative	Postoperative	*p*-Value
Giraffe neck sign	86.7%	20.0%	<0.001 *
Spreading roots sign	73.3%	0%	<0.001 *
Truncation sign	96.7%	16.7%	<0.001 *
White meniscus sign	90.0%	26.7%	<0.001 *

* Significant improvement compared with preoperative value (*p* < 0.05).

**Table 4:** Healing status of the MMPRT based on second-look arthroscopy performed at 1 year postoperatively.

	N	Percentage (%)
Complete healing	11	36.7
Lax healing	10	33.3
Scar healing	6	20.0
Failed	3	10.0

Proportions of each healing type were calculated for the 30 knees included in this study.

**Table 5:** Longitudinal Changes in Anchor Hole Width (AHW) Assessed by Linear Mixed-Effects Models.

	1 Month	3 Months	6 Months	1 Year	*p*-Value
R-anchor					
AP-AHW, mm	2.4 ± 0.8 (1.8 to 5.4)	2.6 ± 1.0 (1.2 to 5.6)	2.3 ± 1.1 (0.9 to 5.2)	1.9 ± 1.1 (0.8 to 6.1)	0.03 *
ML-AHW, mm	2.3 ± 0.6 (1.8 to 4.1)	2.7 ± 1.0 (1.2 to 5.7)	2.3 ± 1.0 (1.0 to 4.9)	2.0 ± 1.0 (0.9 to 4.6)	0.09
M-anchor					
AP-AHW, mm	2.8 ± 0.8 (1.8 to 4.6)	3.7 ± 1.1 (1.9 to 6.2)	3.6 ± 1.3 (1.2 to 6.4)	3.4 ± 1.3 (1.7 to 6.5)	0.18
ML-AHW, mm	2.7 ± 0.7 (1.8 to 4.3)	3.5 ± 0.9 (2.0 to 5.6)	3.3 ± 1.1 (1.5 to 5.7)	3.1 ± 1.1 (1.2 to 5.3)	<0.001 *
MP-anchor					
AP-AHW, mm	2.6 ± 0.6 (1.8 to 4.1)	3.4 ± 1.0 (1.9 to 6.5)	3.4 ± 1.1 (0.9 to 6.1)	3.1 ± 1.1 (0.9 to 6.0)	0.27
ML-AHW, mm	2.3 ± 0.6 (1.8 to 3.9)	3.0 ± 0.8 (1.5 to 4.6)	2.9 ± 0.9 (1.3 to 5.3)	2.7 ± 1.0 (1.2 to 6.0)	0.004 *

All data are presented as mean ± standard deviation (range), unless otherwise indicated. M-anchor: 1.4 mm JuggerKnot Soft Anchor System placed at the midbody; MP-anchor: 1.4 mm JuggerKnot Soft Anchor System placed at the midbody–posterior transition zone; AP-AHW, anteroposterior anchor hole width; ML-AHW, mediolateral anchor hole width; * Significant improvement compared with preoperative value (*p* < 0.05).

**Table 6:** Comparison of AP and ML-AHW between the M-anchor and MP-anchor at 1, 3, 6 months, and 1 year postoperatively.

	M-Anchor	MP-Anchor	*p*-Value
AP-AHW, mm			
1 mo Post-op	2.8 ± 0.8 (1.8 to 4.6)	2.6 ± 0.6 (1.8 to 4.1)	0.21
3 mo Post-op	3.7 ± 1.1 (1.9 to 6.2)	3.4 ± 1.0 (1.9 to 6.5)	0.09
6 mo Post-op	3.6 ± 1.3 (1.2 to 6.4)	3.4 ± 1.1 (0.9 to 6.1)	0.36
1 yr Post-op	3.4 ± 1.3 (1.7 to 6.5)	3.1 ± 1.1 (0.9 to 6.0)	0.10
ML-AHW, mm			
1 mo Post-op	2.7 ± 0.7 (1.8 to 4.3)	2.3 ± 0.6 (1.8 to 3.9)	0.01 *
3 mo Post-op	3.5 ± 0.9 (2.0 to 5.6)	3.0 ± 0.8 (1.5 to 4.6)	0.01 *
6 mo Post-op	3.3 ± 1.1 (1.5 to 5.7)	2.9 ± 0.9 (1.3 to 5.3)	0.01 *
1 yr Post-op	3.1 ± 1.1 (1.2 to 5.3)	2.7 ± 1.0 (1.2 to 6.0)	0.02 *

All data are presented as mean ± standard deviation (range), unless otherwise indicated. M-anchor: 1.4 mm JuggerKnot Soft Anchor System placed at the midbody; MP-anchor: 1.4 mm JuggerKnot Soft Anchor System placed at the midbody–posterior transition zone; AP-AHW, anteroposterior anchor hole width; ML-AHW, mediolateral anchor hole width; 1 mo Post-op, postoperative 1 month; 3 mo Post-op, postoperative 3 months; 6 mo Post-op, postoperative 6 months; 1 yr Post-op, postoperative 1 year. * Significant improvement compared with preoperative value (*p* < 0.05).

**Table 7:** Multivariable associations between anchor hole expansion and postoperative medial meniscus extrusion (MME).

	β	95% CI	*p*-Value
R-anchor			
AP-AHW	0.15	−0.57 to 0.87	0.66
ML-AHW	0.25	−0.38 to 0.87	0.42
M-anchor			
AP-AHW	0.77	0.29 to 1.25	0.003 *
ML-AHW	0.81	0.35 to 1.27	0.002 *
MP -anchor			
AP-AHW	0.23	−0.67 to 1.13	0.6
ML-AHW	0.49	−0.20 to 1.18	0.15

Multivariable linear regression analyses adjusted for age, body mass index, correction angle, bone mineral density, postoperative percentage of mechanical axis (%MA), postoperative mechanical medial proximal tibial angle (mMPTA), postoperative mechanical lateral distal femoral angle (mLDFA), and postoperative joint line convergence angle (JLCA). * Significant increase compared with the immediate postoperative value (*p* < 0.05).

## Data Availability

The original contributions presented in this study are included in the article. Further inquiries can be directed to the corresponding author.
